# The relationship between Stroma AReactive Invasion Front Areas (SARIFA), Warburg-subtype and survival: results from a large prospective series of colorectal cancer patients

**DOI:** 10.1186/s40170-024-00349-z

**Published:** 2024-07-11

**Authors:** Kelly Offermans, Nic G. Reitsam, Colinda C. J. M. Simons, Bianca Grosser, Jessica Zimmermann, Heike I. Grabsch, Bruno Märkl, Piet A. van den Brandt

**Affiliations:** 1 Department of Epidemiology, GROW Research Institute for Oncology and Reproduction, Maastricht University Medical Center+, Maastricht, the Netherlands; 2 Pathology, Medical Faculty, University of Augsburg, Augsburg, Germany; 3 Bavarian Cancer Research Center (BZKF), Augsburg, Germany; 4 Department of Pathology, GROW Research Institute for Oncology and Reproduction, Maastricht University Medical Center+, Maastricht, the Netherlands; 5 Pathology and Data Analytics, Leeds Institute of Medical Research at St James’s, University of Leeds, Leeds, United Kingdom; 6 Department of Epidemiology, Care and Public Health Research Institute (CAPHRI), Maastricht University Medical Center+, Maastricht, the Netherlands

**Keywords:** Colorectal cancer, Biomarker, Histopathology, Tumour metabolism, Invasion front, Tumour microenvironment, Rectal cancer

## Abstract

**Background:**

Stroma AReactive Invasion Front Areas (SARIFA) is a recently identified haematoxylin & eosin (H&E)based histopathologic biomarker in gastrointestinal cancers, including colorectal cancer (CRC), defined as direct contact between tumour cells and adipocytes at the tumour invasion front. The current study aimed at validating the prognostic relevance of SARIFA in a large population-based CRC series as well as at investigating the relationship between SARIFA-status and previously established Warburg-subtypes, both surrogates of the metabolic state of the tumour cells.

**Methods:**

SARIFA-status (positive *versus* negative) was determined on H&E slides of 1,727 CRC specimens. Warburg-subtype (high *versus* moderate *versus* low) data was available from our previous study. The associations between SARIFA-status, Warburg-subtype, clinicopathological characteristics and CRC-specific as well as overall survival were investigated.

**Results:**

28.7% (*n*=496) CRC were SARIFA-positive. SARIFA-positivity was associated with more advanced disease stage, higher pT category, and more frequent lymph node involvement (all *p*<0.001). SARIFA-positivity was more common in Warburg-high CRC. 44.2% (*n*=219) of SARIFA-positive CRCs were Warburg-high compared to 22.8% (*n*=113) being Warburg-low and 33.1% (*n*=164) being Warburg-moderate (p<0.001). In multivariable-adjusted analysis, patients with SARIFA-positive CRCs had significantly poorer CRC-specific (HR_CRC-specific_ 1.65; 95% CI 1.41-1.93) and overall survival (HR_overall survival_ 1.46; 95% CI 1.28-1.67) independent of clinically known risk factors and independent of Warburg-subtype. Combining the SARIFA-status and the Warburg-subtype to a combination score (SARIFA-negative/Warburg-high *versus* SARIFA-positive/Warburg-low *versus* SARIFA-positive/Warburg-high, and so on) did not improve the survival prediction compared to the use of SARIFA-status alone (SARIFA-negative + Warburg-high: HR_CRC-specific_ 1.08; 95% CI 0.84-1.38; SARIFA-positive + Warburg-low: HR_CRC-specific_ 1.79; 95% CI 1.32-2.41; SARIFA-positive + Warburg-high: HR_CRC-specific_ 1.58; 95% CI 1.23-2.04).

**Conclusions:**

Our current study is the by far largest external validation of SARIFA-positivity as a novel independent negative prognostic H&E-based biomarker in CRC. In addition, our study shows that SARIFA-positivity is associated with the Warburg-high subtype. Further research is warranted to provide a more mechanistic understanding of the underlying tumour biology. Based on our data, we conclude SARIFA-status should be implemented in pathologic routine practice to stratify CRC patients.

**Supplementary Information:**

The online version contains supplementary material available at 10.1186/s40170-024-00349-z.

## Introduction

Colorectal cancer (CRC) is the third most common cancer worldwide and contributes substantially to the global burden of disease [1]. In CRC, the conventional radiologic and pathological disease stage according to the tumour-node-metastasis (TNM) classification remains the most important tool for therapeutic decision-making in everyday practice [2]. However, CRC is a heterogeneous disease with different histologic and molecular subtypes that are associated with different outcomes [3–6]. Hence TNM stage as well as other current standard of care histopathological biomarkers, such as tumour budding or grade of differentiation, are insufficient in adequately stratifying CRC patients. Although recently proposed RNAexpression based approaches such as CINSARC [7] or consensus molecular subtypes (CMS) [5] have shown some promising results as potential biomarkers; none of them have been implemented into daily practice as these molecular subtyping approaches rely on technically challenging assays. Hence, there remains an urgent clinical need to better stratify CRC patients using cost-effective, reliable and sensitive biomarkers that can be easily integrated into clinical routine. Ideally, such novel biomarkers are related to the tumour biology and hence represent potential novel therapeutic targets at the same time.

We recently established Stroma AReactive Invasion Front Areas (SARIFA) as a Haematoxylin & Eosin (H&E)based prognostic biomarker in patients with colon or gastric cancer [8, 9]. SARIFA-positivity is defined as direct contact between tumour cells and adipocytes at the tumour invasion front. Our previous study suggested that SARIFA-positivity is associated with upregulation of the lipid metabolism in tumour cells as well as an altered immune response, resulting in a substantial decrease in natural killer (NK) cells in the peripheral blood of SARIFA-positive CRC patients [8–10]. Independent from our own work, several studies using deep learning (DL) algorithms to analyse H&E stained CRC tissue sections identified the colocalization of tumour cells and adipocytes as features with potential prognostic relevance [11–14]. Furthermore, several experimental studies highlighted the key role of adipocytes and lipids in cancer progression in the past [15, 16]. Therefore, it seems likely that SARIFA represents a morphological surrogate of an aggressive tumour biology.

It has been known since the 1920s that tumour cells reprogram their metabolism from oxidative phosphorylation towards aerobic glycolysis. This phenomenon is named after its discoverer Otto Warburg as the Warburg-effect [17], and has been shown to be relevant in CRC [18, 19]. Moreover, it is thought that the Warburg-effect contributes to a more aggressive behaviour and therapy resistance of cancer cells [20, 21]. We have shown previously that immunohistochemistry (IHC)-based Warburg-subtyping, based on the expression of six glycolytic proteins and transcriptional regulators (GLUT1, PKM2, LDHA, MCT4, p53, PTEN), was associated with CRC patient prognosis such that patients with Warburg-high CRCs had the poorest prognosis [22].

Aerobic glycolysis (e.g. the Warburg-effect) as well as upregulation of lipid metabolism are two key interdependent metabolic pathways in cancer progression [23]. Targeting both pathways simultaneously has shown anti-carcinogenic effects in vitro as well as in mice models of different cancer types including colon cancer [24].

To date, no histopathologic biomarker potentially reflecting metabolic changes in tumour cells is used in clinical routine. SARIFA-status and Warburg-subtype can be reliably assessed on tumour tissue sections using H&E staining and IHC, respectively, e.g. with methods which are already routinely used in the histopathology laboratory. The current study aimed (i) to validate the prognostic value of SARIFA-status in a large population-based series of CRC patients, and (ii) to explore the association between Warburg-subtype and SARIFA-status.

## Methods

### Design and study population

The population-based series of colorectal cancer (CRC) patients was obtained from the prospective Netherlands Cohort Study on diet and cancer (NLCS), which has been described previously [25]. The NLCS was initiated in September 1986 and included 120,852 men and women aged 55 to 69 years [25]. At study baseline, participants completed a mailed, self-administered questionnaire on their dietary habits and other cancer risk factors [25].

**Fig. 1:**
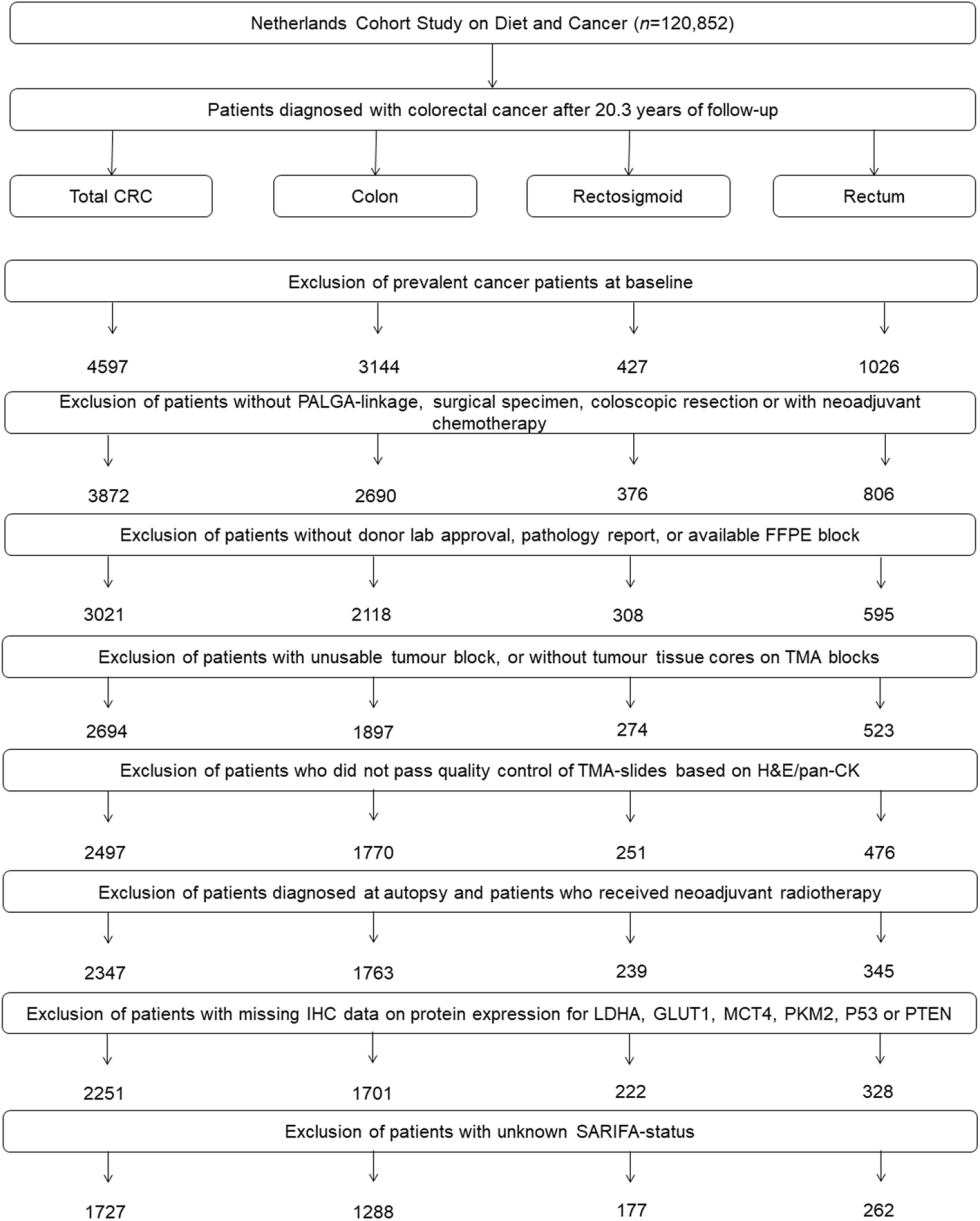
Flow diagram of the number of colorectal cancer patients available for analyses in the Netherlands Cohort Study (NLCS), 1986-2006. CRC, colorectal cancer; PALGA, Netherlands pathology database; TMA, tissue microarray

The entire cohort was followed-up for cancer incidence by annual record linkage with the Netherlands Cancer Registry and PALGA, the nationwide Dutch Pathology Registry [26], covering 20.3 years of follow-up (September 17, 1986 until January 1, 2007). The completeness of cancer incidence follow-up was estimated to be >96% [27]. After excluding patients with a history of cancer (excluding non-melanoma skin cancer) at baseline, 4,597 incident CRC patients were available (Fig. 1).

The NLCS was approved by the institutional review boards of the TNO Quality of Life Research Institute (Zeist, the Netherlands) and Maastricht University (Maastricht, the Netherlands). All cohort members consented to participate in this study by completing the questionnaire. Ethical approval was obtained from the Medical Ethical Committee (METC) of Maastricht University Medical Center+ (Maastricht, the Netherlands).

### Clinical characteristics and follow-up

Data on patient and tumour characteristics, including age at diagnosis, pathological tumour-node-metastasis (pTNM) stage, tumour location, tumour differentiation grade, adjuvant therapy and survival were collected for a previous study [22]. Cause of death was retrieved from Statistics Netherlands. Vital status was available for 2,346 CRC patients, and information of CRC-specific death was available for 2,309 patients.

### Warburg-subtyping

Warburg-subtype and mismatchrepair (MMR) status were determined in a previous study (see [22] for details). In short, tissue microarray sections of the Rainbow-Tissue MicroArray (TMA) project [28] were subjected to immunohistochemistry (IHC) for proteins related to the Warburg-effect (LDHA, GLUT1, MCT4, PKM2, p53, PTEN) and MMRrelated proteins (MLH1, MSH2) [22]. After excluding patients with missing protein expression data, 2,251 CRC patients were categorised as “Warburg-low” (*n*=652, 29.0%), “Warburg-moderate” (*n*=802, 35.6%) or “Warburg-high” (*n*=797, 35.4%; Fig. 1) [22]. Tumours with loss of either MLH1 or MSH2 expression were categorised as MMR deficient (dMMR) [22].

### Assessment of SARIFA-status

SARIFA-status was assessed on digitised H&E-stained whole slide images (WSIs) according to our previous publications on SARIFA in CRC [9, 10]. SARIFA-positivity was defined as an area at the tumour invasion front where at least one tumour gland or at least a group of ≥5 tumour cells were directly adjacent to adipocytes, without intervening inflammatory infiltrate or desmoplastic stroma reaction. The presence of one such area was sufficient to categorise a CRC as SARIFA-positive. In the absence of such an area, the CRC was categorised as SARIFA-negative. We have previously demonstrated a low interobserver variability (for CRC: kappa up to 0.87) for the assessment of SARIFA-status on H&E-stained resection specimens [8, 9]. All CRCs were classified by JZ and/or NGR, both being appropriately trained to establish the SARIFA-status, and supervised by BM and HG, both senior pathologists.

From all NLCS CRC resection specimens, a single tumour containing H&Estained tissue section had been scanned previously at 40x magnification (Aperio XT whole slide scanner, Aperio Technologies, Vista, CA, USA). Digital slides were accessed using QuPath [29] for SARIFA-status assessment. Slides were excluded from SARIFA-status assessment if the overall scanning quality was inferior, only superficial tumour parts were present or absence of any tumour on slide. If tumour cells were seen directly adjacent to adipocytes in the submucosa in pT2 CRCs, these were classified as SARIFA-positive. In pT3/4 CRCs, the SARIFA-status was established on the basis of tumour cells in the pericolonic adipose tissue, the SARIFA-status in the submucosa was not considered in pT3/4 CRCs.

**Fig. 2:**
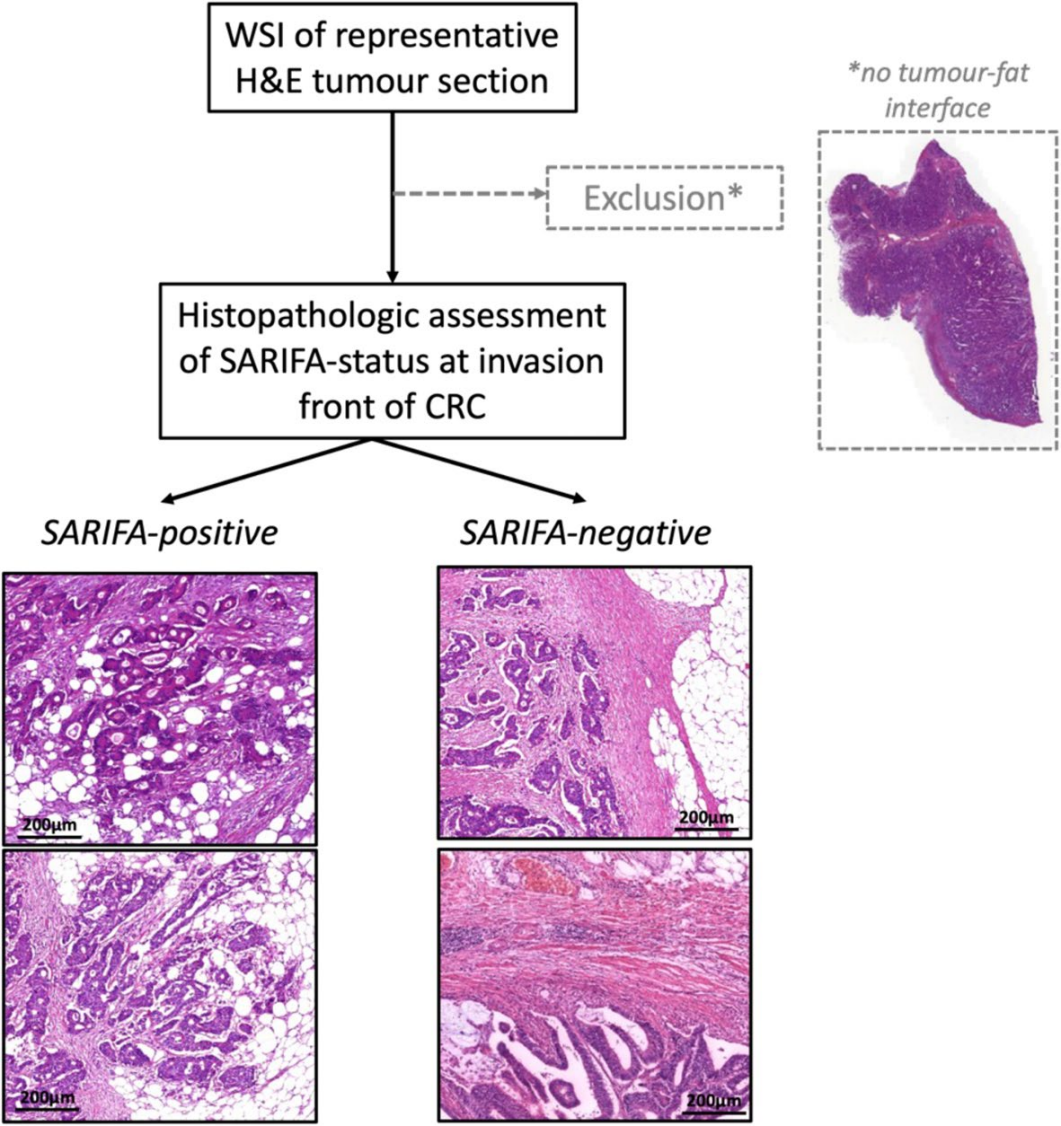
Histopathological assessment of SARIFA-status. Digitised WSI of CRC patients within NLCS were screened for suitable cases. Cases that only depicted superficial tumour parts and not the tumour-fat interface, which is necessary for SARIFA-classification, were excluded (*). Other reasons for exclusion (*) were inferior scanning quality (folds etc.), a fragmented invasion front or only normal colonic/rectal mucosa on the slide. SARIFA-status is a solely H&Ebased biomarker, which is characterised by the direct contact between adipocytes and tumour cells at the invasion front (SARIFA-positive), and was scored as described previously [10]. If there was inflammation or desmoplasia between tumour cells and adipocytes, cases were classified as SARIFA-negative. CRC, colorectal cancer; H&E, haematoxylin and eosin; NLCS, Netherlands Cohort Study; SARIFA, Stroma AReactive Invasion Front Area; WSI, whole slide image. Scale bar: 200µm

Histopathological assessment of SARIFA-status is illustrated in Fig. 2.

### Statistical analyses

Descriptive statistics and frequency distributions for the total series of CRC patients, as well as stratified by SARIFA-status, were calculated for clinical and molecular characteristics. Differences between patients according to SARIFA-status were evaluated using Chi-square tests (categorical variables) or Kruskal-Wallis tests (continuous variables).

The primary endpoints of the current study were CRC-specific survival, defined as the time from CRC diagnosis to CRC-related death or end of follow-up, and overall survival, defined as the time from CRC diagnosis to death from any cause or end of follow-up. Survival analyses were restricted to 10 years of follow-up.

The relationship between SARIFA-status and CRC-specific or overall survival was estimated using Kaplan-Meier curves and Wilcoxon tests. Hazard ratios (HRs) and 95% confidence intervals (CIs) were estimated with Cox proportional hazards regression. The proportional hazards assumption was tested using the scaled Schoenfeld residuals [30], by evaluating -log-log transformed survival curves, and by introducing time-covariate interactions into the models. For Cox regression analyses, a separate category ‘unknown’ was used for patients with unknown SARIFA-status (*n* = 524), to enable inclusion of these patients in the Cox proportional hazards models.

HRs were adjusted for a set of a priori selected prognostic factors: age at diagnosis (years), sex (men, women), tumour location (colon, rectosigmoid, rectum), pTNM stage (I, II, III, IV), differentiation grade (well, moderate, poor/undifferentiated), MMR status (pMMR, dMMR), and adjuvant therapy (no, yes). A separate category (‘unknown’) was used for patients with unknown information regarding clinical characteristics, such as pTNM stage, grade of differentiation, adjuvant therapy, or MMR status, to enable inclusion of these patients in the Cox proportional hazards models. Additionally, analyses stratified on tumour location and pTNM stage were performed.

To investigate whether SARIFA-status and Warburg-subtype were independent prognostic markers, multivariable-adjusted models were mutually adjusted for Warburg-subtype and SARIFA-status. Furthermore, additional stratified analyses were performed to investigate (1) the association between SARIFA-status and survival according to Warburg-subtype, and (2) the association between Warburg-subtype and survival according to SARIFA-status. Moreover, SARIFA-status and Warburg-subtype were combined into a combination score: SARIFA-negative/Warburg-low *versus* SARIFA-negative/Warburg-moderate *versus* SARIFA-negative/Warburg-high *versus* SARIFA-positive/Warburg-low *versus* SARIFA-positive/Warburg-moderate *versus* SARIFA-positive/Warburg-high for survival analyses.

Cancer stage was based on the TNM classification edition that was valid at the time of cancer diagnosis. Hence, five different TNM versions have been used during the total follow-up period (TNM versions 3-6; Supplementary Table S1). The main TNM stage groupings (I, II, III, IV), however, remained essentially unchanged [31]. Year of diagnosis (per 3 years) and TNM version (3, 4.1, 4.2, 5, 6) were considered potential confounders, and were retained in the final models if they introduced a ≥10% change in HRs.

## Results

### Study population

Warburg-subtype data was available for 2,251 colorectal cancer (CRC) patients from the Netherlands Cohort Study (NLCS). Patients were previously classified as Warburg-low (*n*=652, 29.0%), Warburg-moderate (*n*=802, 35.6%), or Warburg-high (*n*=797, 35.4%) based on the combined protein expression levels of LDHA, GLUT1, MCT4, PKM2, p53 and PTEN [22]. During the current study, SARIFA-status was established for 1,727 patients of which 1,231 (54.7%) were classified as SARIFA-negative, 496 (22.0%) as SARIFA-positive and 524 (23.3%) as SARIFA-unknown (see methods section). Warburg-subtype and SARIFA-status were available for 1,727 (76.7%) CRC patients.

### Relationship between SARIFA-status and clinical characteristics

Clinical characteristics of the 1,727 incident CRC patients with complete information regarding Warburg-subtype and SARIFA-status are presented in Table 1. Patients with SARIFA-positive CRC more frequently had cancers located in the colon compared to patients with SARIFA-negative CRC (85.7% versus 70.1%, *p*<0.001). Patients with SARIFA-positive CRC presented more frequently with an advanced disease stage (pTNM III-IV, *p*<0.001), increased depth of invasion (pT3-4, *p*<0.001), increased number of lymph nodes with metastasis (pN+, *p*<0.001) and more frequently had poorly or undifferentiated cancers compared to patients with SARIFA-negative CRC (29.8% *versus* 13.3%, *p*<0.001). Lastly, due to the higher pTNM stage, patients with SARIFA-positive CRC were more often treated with adjuvant therapy compared to patients with SARIFA-negative CRC (22.2% versus 14.0%, *p*<0.001). No significant differences in clinical characteristics were observed between SARIFA-known (SARIFA-positive and SARIFA-negative, *n*=1,727) and SARIFA-unknown (*n*=524) CRC patients (Supplementary Table S2), proving that our selection criteria were unbiased.

**Table 1:** Clinical and molecular characteristics of the total series of colorectal cancer patients within the Netherlands Cohort Study (NLCS; 1986-2006), as well as according to SARIFA-status (SARIFA-positive and SARIFA-negative)

	**Total series of CRC patients** **(*n* = 1,727)**	**SARIFA-status**		
		**Negative (*n* = 1,231)**	**Positive (*n* = 496)**	***p-*value**^**a**^
**Age at diagnosis in years, median (range)**	74.0 (55.0-89.0)	74.0 (55.0-89.0)	74.0 (55.0-88.0)	0.355^b^
**Sex, *n* (%)**				
Men	958 (55.5)	694 (56.4)	264 (53.2)	0.233
Women	769 (44.5)	537 (43.6)	232 (46.8)	
**Tumour location, *n* (%)**				
Colon	1288 (74.6)	863 (70.1)	425 (85.7)	<0.001
Rectosigmoid	177 (10.3)	142 (11.5)	35 (7.1)	
Rectum	262 (15.2)	226 (18.4)	36 (7.3)	
**pTNM stage, *n* (%)**				
I	315 (18.2)	309 (25.1)	6 (1.2)	<0.001
II	672 (38.9)	514 (41.8)	158 (31.9)	
III	454 (26.3)	277 (22.5)	177 (35.7)	
IV	245 (14.2)	103 (8.4)	142 (28.6)	
Unknown	41 (2.4)	28 (2.3)	13 (2.6)	
**Tumour extension (pT), *n* (%)**				
T1	65 (3.8)	65 (5.3)	-	<0.001
T2	306 (17.7)	297 (24.1)	9 (1.8)	
T3	1138 (65.9)	764 (62.1)	374 (75.4)	
T4	174 (10.1)	76 (6.2)	98 (19.8)	
Unknown	44 (2.6)	29 (2.4)	15 (3.0)	
**Lymph node involvement (pN), *n* (%)**				
N0	900 (52.1)	731 (59.4)	169 (34.0)	<0.001
N+	629 (36.4)	344 (27.9)	285 (57.5)	
Unknown	198 (11.5)	156 (12.7)	42 (8.5)	
**Differentiation grade, *n* (%)**				
Well	152 (8.8)	125 (10.2)	27 (5.4)	<0.001
Moderate	1138 (65.9)	845 (68.6)	293 (59.1)	
Poor/undifferentiated	312 (18.1)	164 (13.3)	148 (29.8)	
Unknown	125 (7.2)	97 (7.9)	28 (5.7)	
**Adjuvant therapy, *n* (%)**				
No	1426 (82.6)	1046 (85.0)	380 (76.6)	<0.001
Yes	282 (16.3)	172 (14.0)	110 (22.2)	
Unknown	19 (1.1)	13 (1.1)	6 (1.2)	
**MMR status, *n* (%)**				
Proficient	1537 (89.0)	1086 (88.2)	451 (90.9)	0.104
Deficient	190 (11.0)	145 (11.8)	45 (9.1)	
Unknown	-	-	-	

^a^*P*-value for the Chi-square test, unless otherwise specified. Presented *p*-values exclude the ‘unknown’ category for pTNM stage, pT, pN, differentiation grade, adjuvant therapy, and MMR status.

^b^*P*-value for the Kruskal-Wallis test

### Relationship between Warburg-subtype and SARIFA-status

The Warburg-subtype was previously established and was based on immunohistochemistry (IHC) of six proteins related to the Warburg-effect (i.e., GLUT1, PKM2, LDHA, MCT4, p53, PTEN) [22]. Expression levels of these Warburg-related proteins (low/moderate/high) were combined into a pathway-based sum score, ranging from 0 to 12, whereby a higher sum score indicated a higher probability of the presence of the Warburg-effect [22]. Based on this sum score, CRC patients were classified as Warburg-low (sum score 0-3), Warburg-moderate (sum score 4-5), or Warburg-high (sum score 6-12) [22].

**Fig. 3:**
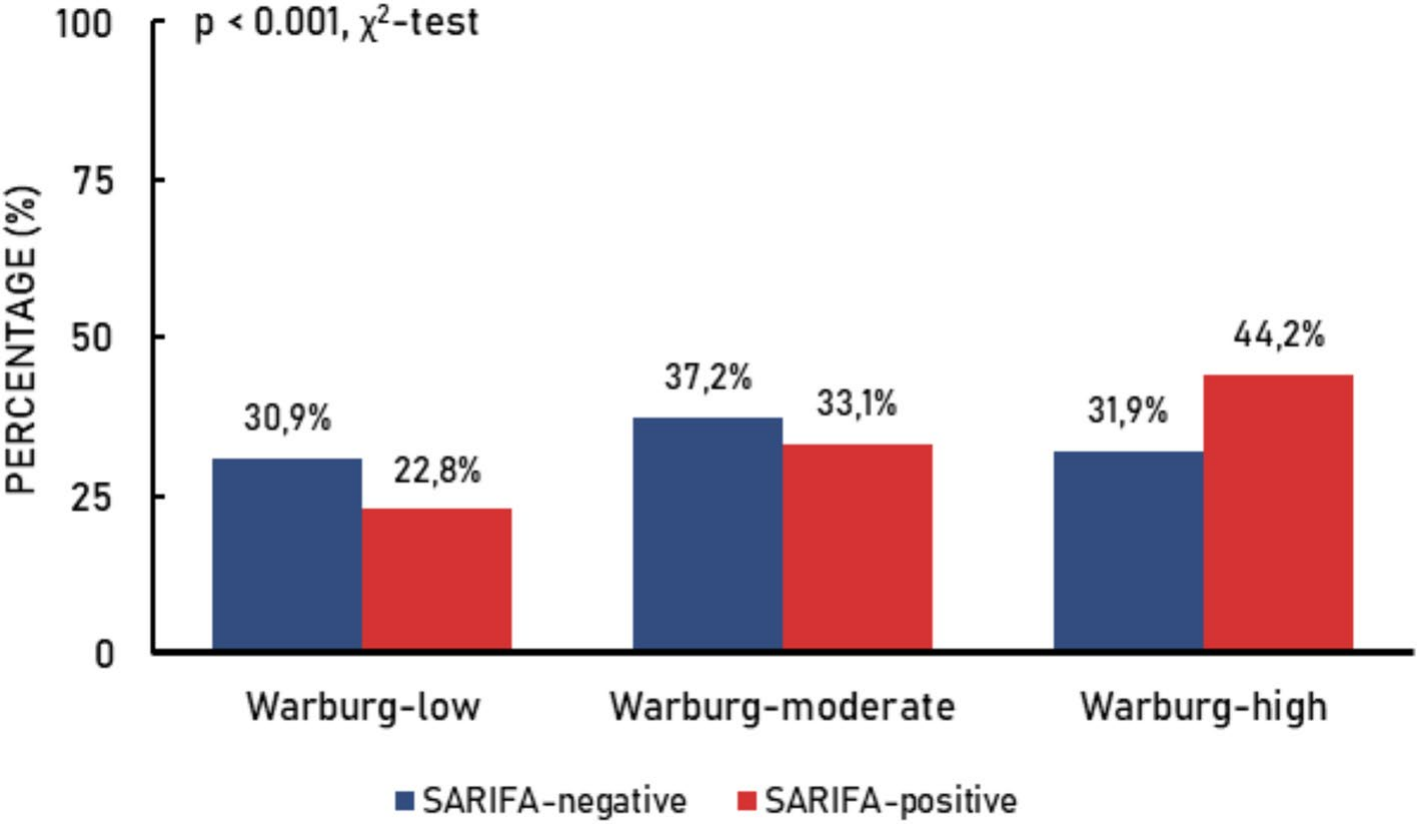
Relationship between SARIFA-status (SARIFA-negative, SARIFA-positive) and Warburg-subtype (Warburg-low, Warburg-moderate, Warburg-high) for colorectal cancer patients (*n*=1,727) within the Netherlands Cohort Study (NLCS; 1986-2006)

For the current study, the relationship between SARIFA-status and Warburg-subtype was investigated in 1,727 CRC patients. There was a significant relationship between SARIFA-status and Warburg-subtype (*p*<0.001). Within the group of SARIFA-positive CRC, 219 (44.2%) were Warburg-high, 164 (33.1%) Warburg-moderate and 113 (22.8%) Warburg-low (Fig. 3). In contrast, within the group of SARIFA-negative CRC, the frequency of the Warburg-subtypes was almost equally distributed (*n*=380 (30.9%) Warburg-low, *n*=458 (37.2%) Warburg-moderate, *n*=393 (31.9%) Warburg-high; Fig. 3).

### Relationship between SARIFA-status, Warburg-subtype and survival

The median (range) follow-up time since diagnosis was 4.79 years (0.0027 – 25.99 years). Survival analyses were restricted to 10 years of follow-up. During these first 10 years of follow-up, 1,463 deaths were observed, of which 933 (63.8%) were CRC-related deaths.

**Fig. 4:**
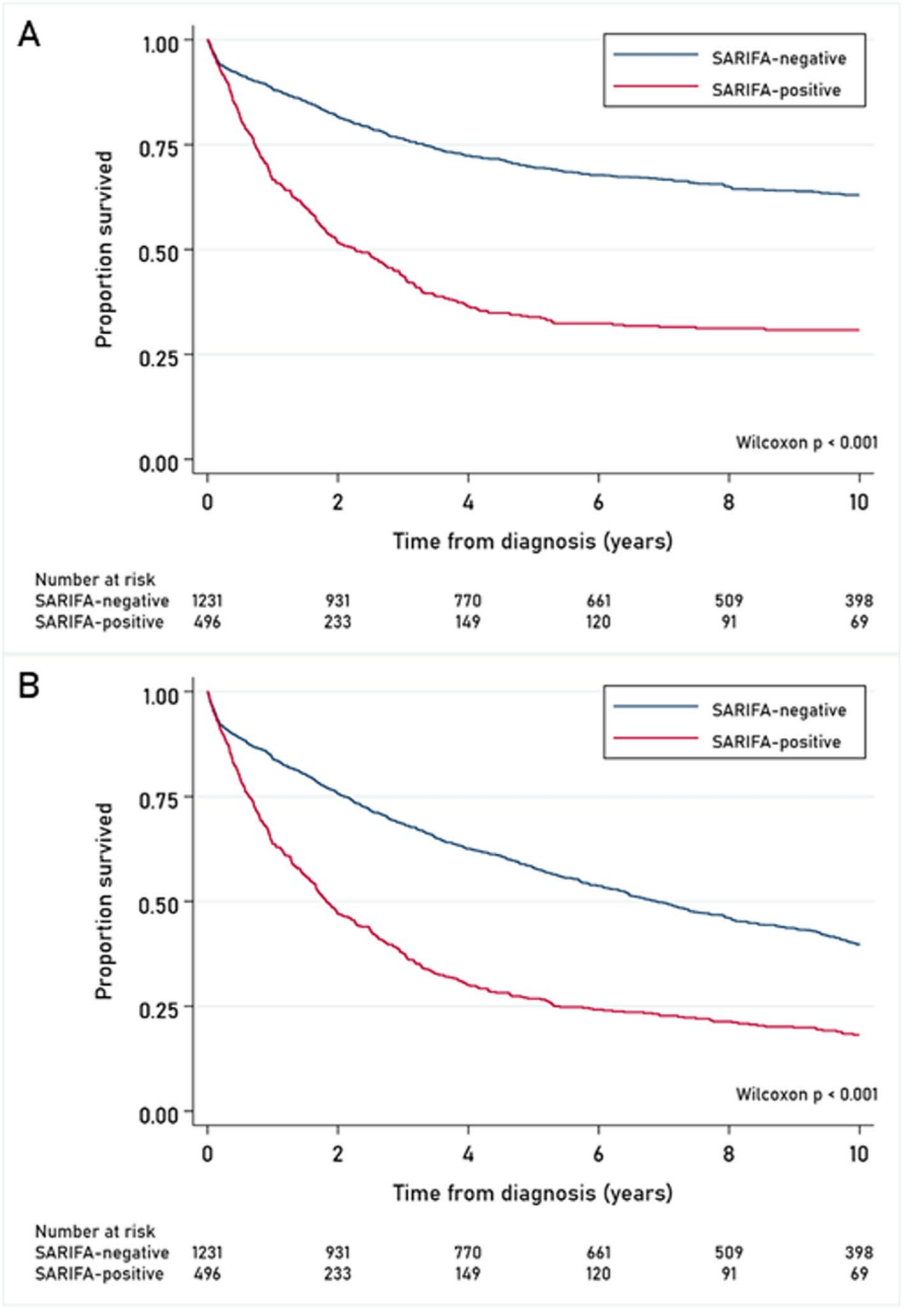
Univariable Kaplan-Meier curves showing (**A**) CRC-specific survival and (**B**) overall survival of 1,727 colorectal cancer patients within the Netherlands Cohort Study (NLCS; 1986-2006) according to SARIFA status (SARIFA-positive and SARIFA-negative)

Univariable Kaplan-Meier curves showed significant differences in CRC-specific survival (*p*<0.001) and overall survival (*p*<0.001) according to SARIFA-status (Fig. 4). Patients with SARIFA-positive CRC had a poorer CRC-specific (HR_CRC-specific_ 2.75; 95% CI 2.37-3.19) and overall survival (HR_overall_ 2.09; 95% CI 1.85-2.37) (Table 2).

**Table 2:** Univariable and multivariable-adjusted hazard ratios (HRs) and 95% confidence intervals (CIs) for associations between the SARIFA-status and CRC-specific and overall survival of colorectal cancer patients (n = 2,251) within the Netherlands Cohort Study (NLCS, 1986-2006)

	**N**	**CRC-specific survival**				**Overall survival**			
		**CRC deaths (%)**	**HR (95% CI)**			**Deaths (%)**	**HR (95% CI)**		
			**Univariable**	**Multivariable-adjusted**^**a**^	**Multivariable-adjusted**^c^		**Univariable**	**Multivariable-adjusted**^a^	**Multivariable-adjusted**^c^
**SARIFA**									
SARIFA-negative	1231	406 (33.0)	1.00 (ref)	1.00 (ref)	1.00 (ref)	726 (59.0)	1.00 (ref)	1.00 (ref)	1.00 (ref)
SARIFA-positive	496	317 (63.9)	2.75 (2.37-3.19)	1.67 (1.43-1.95)	1.65 (1.41-1.93)	402 (81.0)	2.09 (1.85-2.37)	1.49 (1.30-1.69)	1.46 (1.28-1.67)
Unknown	524	210 (40.1)	1.35 (1.15-1.60)	1.22 (1.03-1.45)	1.22 (1.03-1.45)	335 (63.9)	1.22 (1.07-1.39)	1.15 (1.01-1.32)	1.15 (1.01-1.32)
**Warburg-subtype**									
Warburg-low	652	245 (37.6)	1.00 (ref)	1.00 (ref)^b^	1.00 (ref)	402 (61.7)	1.00 (ref)	1.00 (ref)^b^	1.00 (ref)
Warburg-moderate	802	339 (42.3)	1.16 (0.98-1.36)	1.04 (0.88-1.22)^b^	1.03 (0.87-1.22)	514 (64.1)	1.07 (0.94-1.22)	1.01 (0.89-1.15)^b^	1.01 (0.88-1.15)
Warburg-high	797	349 (43.8)	1.27 (1.08-1.50)	1.17 (0.99-1.38)^b^	1.10 (0.93-1.30)	547 (68.6)	1.24 (1.09-1.41)	1.18 (1.03-1.34)^b^	1.14 (1.00-1.30)
**Age at diagnosis (per year)**	2251	933 (41.4)	1.01 (1.00-1.03)	1.02 (1.01-1.03)	1.02 (1.01-1.03)	1463 (65.0)	1.04 (1.03-1.05)	1.05 (1.04-1.06)	1.05 (1.04-1.06)
**Sex**									
Men	1242	515 (41.5)	1.00 (ref)	1.00 (ref)	1.00 (ref)	846 (68.1)	1.00 (ref)	1.00 (ref)	1.00 (ref)
Women	1009	418 (41.4)	0.98 (0.86-1.11)	0.96 (0.84-1.10)	0.96 (0.84-1.09)	617 (61.1)	0.87 (0.78-0.97)	0.81 (0.73-0.90)	0.81 (0.72-0.90)
**Tumour location**									
Colon	1701	714 (42.0)	1.00 (ref)	1.00 (ref)	1.00 (ref)	1120 (65.8)	1.00 (ref)	1.00 (ref)	1.00 (ref)
Rectosigmoid	222	82 (36.9)	0.85 (0.68-1.07)	0.99 (0.79-1.25)	0.99 (0.79-1.25)	147 (66.2)	0.97 (0.82-1.16)	1.14 (0.95-1.35)	1.14 (0.95-1.36)
Rectum	328	137 (41.8)	0.91 (0.76-1.09)	1.23 (1.02-1.49)	1.23 (1.02-1.49)	196 (59.8)	0.81 (0.70-0.95)	1.07 (0.92-1.26)	1.08 (0.92-1.26)
**pTNM stage**									
I	424	67 (15.8)	1.00 (ref)	1.00 (ref)	1.00 (ref)	194 (45.8)	1.00 (ref)	1.00 (ref)	1.00 (ref)
II	868	237 (27.3)	1.86 (1.42-2.44)	1.76 (1.34-2.32)	1.75 (1.33-2.31)	485 (55.9)	1.34 (1.14-1.59)	1.23 (1.04-1.47)	1.23 (1.03-1.46)
III	582	306 (52.6)	4.64 (3.56-6.05)	4.19 (3.17-5.54)	4.20 (3.18-5.55)	425 (73.0)	2.38 (2.01-2.82)	2.18 (1.82-2.62)	2.19 (1.82-2.63)
IV	325	296 (91.1)	18.97 (14.44-24.91)	16.95 (12.69-22.64)	16.92 (12.67-22.61)	323 (99.4)	8.64 (7.17-10.42)	7.97 (6.51-9.77)	7.98 (6.51-9.78)
Unknown	52	27 (51.9)	3.99 (2.55-6.23)	4.03 (2.46-6.60)	4.00 (2.44-6.54)	36 (69.2)	1.92 (1.34-2.73)	2.35 (1.59-3.49)	2.33 (1.57-3.44)
**Differentiation grade**									
Well	201	65 (32.3)	1.00 (ref)	1.00 (ref)	1.00 (ref)	110 (54.7)	1.00 (ref)	1.00 (ref)	1.00 (ref)
Moderate	1478	574 (38.8)	1.29 (1.00-1.67)	1.04 (0.80-1.34)	1.03 (0.79-1.33)	935 (63.3)	1.27 (1.04-1.55)	1.10 (0.90-1.34)	1.08 (0.89-1.32)
Poor/undifferentiated	397	222 (55.9)	2.44 (1.85-3.22)	1.61 (1.21-2.14)	1.58 (1.19-2.11)	305 (76.8)	2.09 (1.68-2.60)	1.53 (1.22-1.92)	1.49 (1.19-1.88)
Unknown	175	72 (41.1)	1.42 (1.01-1.98)	1.05 (0.75-1.48)	1.05 (0.74-1.47)	113 (64.6)	1.35 (1.04-1.75)	1.11 (0.85-1.45)	1.10 (0.84-1.43)
**Adjuvant therapy**									
No	1874	714 (38.1)	1.00 (ref)	1.00 (ref)	1.00 (ref)	1191 (63.6)	1.00 (ref)	1.00 (ref)	1.00 (ref)
Yes	356	209 (58.7)	1.58 (1.35-1.84)	0.75 (0.64-0.89)	0.75 (0.64-0.89)	258 (72.5)	1.21 (1.06-1.39)	0.75 (0.64-0.86)	0.75 (0.65-0.87)
Unknown	21	10 (47.6)	1.23 (0.66-2.29)	1.18 (0.59-2.36)	1.19 (0.60-2.37)	14 (66.7)	1.04 (0.61-1.76)	1.26 (0.71-2.23)	1.26 (0.71-2.24)
**MMR deficiency**									
No	1975	855 (43.3)	1.00 (ref)	1.00 (ref)	1.00 (ref)	1301 (65.9)	1.00 (ref)	1.00 (ref)	1.00 (ref)
Yes	253	68 (26.9)	0.56 (0.44-0.72)	0.55 (0.43-0.72)	0.55 (0.43-0.72)	147 (58.1)	0.78 (0.66-0.93)	0.75 (0.62-0.90)	0.74 (0.62-0.89)
Unknown	23	10 (43.5)	1.06 (0.57-1.99)	1.13 (0.60-2.14)	1.13 (0.60-2.15)	15 (65.2)	1.05 (0.63-1.74)	1.11 (0.66-1.87)	1.10 (0.65-1.85)

*CRC* Colorectal cancer, *HR* Hazard ratio, *CI* Confidence interval

^a^Multivariable-adjusted model included SARIFA status (positive, negative, unknown), age at diagnosis (years), sex (men, women), tumour location (colon, rectosigmoid, rectum), pTNM stage (I, II, III, IV, unknown), differentiation grade (well, moderate, poor/undifferentiated, unknown), adjuvant therapy (no, yes, unknown), MMR deficiency (no, yes, unknown), unless otherwise specified

^b^Multivariable-adjusted model included Warburg-subtype (Warburg-low, Warburg-moderate, Warburg-high), age at diagnosis (years), sex (men, women), tumour location (colon, rectosigmoid, rectum), pTNM stage (I, II, III, IV, unknown), differentiation grade (well, moderate, poor/undifferentiated, unknown), adjuvant therapy (no, yes, unknown), and MMR deficiency (no, yes, unknown)

^c^Multivariable-adjusted model included SARIFA status (positive, negative, unknown), Warburg-subtypes (Warburg-low, Warburg-moderate, Warburg-high), age at diagnosis (years), sex (men, women), tumour location (colon, rectosigmoid, rectum), pTNM stage (I, II, III, IV, unknown), differentiation grade (well, moderate, poor/undifferentiated, unknown), adjuvant therapy (no, yes, unknown), MMR deficiency (no, yes, unknown)

After adjusting for a priori defined confounders (already established risk factors), both SARIFA-status and Warburg-subtype remained significant predictors of survival. Patients with SARIFA-positive CRC had poorer CRC-specific (HR_CRC-specific_ 1.67; 95% CI 1.43-1.95) and overall survival (HR_overall_ 1.49; 95% CI 1.30-1.69) (Table 2). As previously published, patients with Warburg-high CRC had poorer CRC-specific (HR_CRC-specific_ 1.17; 95% CI 0.99-1.38) and overall survival (HR_overall_ 1.18; 95% CI 1.03-1.34) compared to patients with Warburg-low CRC (Table 2). Mutual adjustment for Warburg-subtype and SARIFA-status did not significantly alter observed associations with survival for SARIFA-status (Table 2). To investigate whether combining both markers may lead to an even better patient stratification, we established a combination score (Table 3). Combining both markers (SARIFA-status [negative or positive] + Warburg-subtype [low, moderate or high]) into six categories did not improve patient stratification for survival prediction (SARIFA-negative + Warburg-high: HR_CRC-specific_ 1.08; 95% CI 0.84-1.38; SARIFA-positive + Warburg-low: HR_CRC-specific_ 1.79; 95% CI 1.32-2.41; SARIFA-positive + Warburg-high: HR_CRC-specific_ 1.58; 95% CI 1.23-2.04). This is line with the findings that the prognostic relevance of Warburg-high status decreases substantially when adjusting for SARIFA-status in the multivariable-adjusted model (HR_CRC-specific_ 1.10; 95% CI 0.93-1.30).

**Table 3:** Univariable and multivariable-adjusted hazard ratios (HRs) and 95% confidence intervals (CIs) for associations between the combination score of SARIFA/Warburg-status and CRC-specific and overall survival of colorectal cancer patients (*n* = 1,727) within the Netherlands Cohort Study (NLCS, 1986-2006)

	**N**	**CRC-specific survival**			**Overall survival**		
		**CRC deaths (%)**	**HR (95% CI)**		**Deaths (%)**	**HR (95% CI)**	
			**Univariable**	**Multivariable-adjusted**^**a**^		**Univariable**	**Multivariable-adjusted**^**a**^
**Combination score**							
SARIFA-negative + Warburg-low	380	121 (31.8)	1.00 (ref)	1.00 (ref)	218 (57.4)	1.00 (ref)	1.00 (ref)
SARIFA-negative + Warburg-moderate	458	156 (34.1)	1.07 (0.84-1.35)	0.99 (0.78-1.26)	269 (58.7)	1.02 (0.85-1.22)	1.00 (0.83-1.19)
SARIFA-negative + Warburg-high	393	129 (32.8)	1.07 (0.83-1.37)	1.08 (0.84-1.38)	239 (60.8)	1.11 (0.92-1.33)	1.11 (0.93-1.34)
SARIFA-positive + Warburg-low	113	70 (61.9)	2.58 (1.92-3.46)	1.79 (1.32-2.41)	88 (77.9)	1.93 (1.50-2.47)	1.55 (1.20-1.99)
SARIFA-positive + Warburg-moderate	164	114 (69.5)	3.38 (2.62-4.37)	1.82 (1.40-2.36)	136 (82.9)	2.43 (1.96-3.02)	1.57 (1.26-1.96)
SARIFA-positive + Warburg-high	219	133 (60.7)	2.70 (1.13-1.77)	1.58 (1.23-2.04)	178 (81.3)	2.14 (1.76-2.62)	1.51 (1.23-1.85)

^a^Multivariable-adjusted model included age at diagnosis (years), sex (men, women), tumour location (colon, rectosigmoid, rectum), pTNM stage (I, II, III, IV, unknown), differentiation grade (well, moderate, poor/undifferentiated, unknown), adjuvant therapy (no, yes, unknown), and MMR deficiency (no, yes, unknown)

In line with these findings, stratified analyses (Supplementary Tables S3-S6) showed that SARIFA-positivity was associated with poorer CRC-specific and overall survival in patients with cancers located in the colon, or rectosigmoid (Supplementary Table S3), irrespective of pTNM stage (Supplementary Table S4) or Warburg-subtype (Supplementary Table S5). Interestingly, the Warburg-high subtype was only associated with CRC-specific and overall survival in the subgroup of patients with unknown SARIFA-status (Supplementary Table S6).

## Discussion

In this large prospective series of colorectal cancer (CRC) patients, we (i) investigated the prognostic value of the Stroma AReactive Invasion Front Areas (SARIFA)-status (defined as the direct contact between a tumour gland/tumour cell cluster (≥5 cells) and adipocytes at the invasion front) and (ii) explored the relationship between Warburg-subtype (based on the expression of six glycolytic proteins and transcriptional regulators (GLUT1, PKM2, MCT4, PKM2, p53, PTEN), and H&Ebased SARIFA-status.

We found that patients with SARIFA-positive CRC had a significantly poorer CRC-specific and overall survival compared to patients with SARIFA-negative CRC, independent of known prognostic factors such as disease stage. This association was particularly true for patients with cancers located in the colon and rectosigmoid. However, SARIFA-positivity in early CRCs (pTNM stage I) is very rare, and occurs mainly in the presence of abundant submucosal adipose tissue. Furthermore, our results suggest a relationship between SARIFA-positive CRC and the presence of a Warburg-like metabolic phenotype (i.e. the Warburg-high subtype). Interestingly, both, SARIFA-status and Warburg-subtype, showed independent prognostic value (even though the prognostic value of Warburg-subtype here was lower than that of SARIFA-status).

### SARIFA-status and survival

The results of the current study such as the frequency of SARIFA-positivity and its relationship with survival are consistent with our previous study [9], validating the prognostic value of the SARIFA-status in an independent large prospective cohort of CRC patients. Whereas our previous study only included patients with colon cancer [9], the current study also included cancers located in the rectosigmoid and rectum. Whilst findings from the current study suggest that the relationship between SARIFA-status and survival might also be true for cancers of the rectosigmoid, we did not observe any relationship for rectal cancers. Frequency of SARIFA-positivity gradually decreases from colon (33%) to rectosigmoid (20%) to the rectum (13%). The number of SARIFA-positive rectal cancers (n=36) was limited and while we could observe a significant association between SARIFA-positivity and poorer CRC-specific and overall survival in univariable analysis, this association did not remain significant in multivariable-adjusted analysis. Larger cohorts of rectal cancer patients are necessary to determine the prognostic value of SARIFA-status in rectal cancer, as it is already known that rectal cancers show a different tumour biology compared to tumours in the colon [32]. Here, it is important to raise the question whether the frequency of SARIFA-positive rectal cancer cases is potentially higher in modern cohorts due to improved surgical techniques such as more extensive resection of the mesorectum [33].

### Relationship between SARIFA-status and Warburg-subtyping

It has previously been suggested that the metabolic cross-talk between adipocytes in the tumour microenvironment (TME) and cancer cells may play a pivotal role in cancer progression, by regulating glucose metabolism and promoting the Warburg-effect [34–36]. To the best of our knowledge, our study is the first to investigate the relationship between the Warburg-effect and the SARIFA-status in a large prospective series of incident CRC patients. We found a significant relationship between SARIFA-status and Warburg-subtype and observed that cancers of patients with SARIFA-positive CRC were more frequently Warburg-high, suggesting a potential interplay between these factors.

The potential biological and mechanistic foundation of this association between SARIFA-positivity and Warburg-high status may be a close interconnection between the Warburg-effect and an altered lipid metabolism, as increased glycolysis is necessary for an upregulation of lipid synthesis [23]. Additionally, one could speculate whether the Warburg-effect plays a causally relevant role in the formation of SARIFA, as it is known that the Warburg-effect exerts influence on the tumour microenvironment (TME) by reprogramming neighbouring host cells (e.g., endothelial cells, fibroblasts, immune cells, adipocytes) [37–39]. Due to this close metabolic cross-talk between tumour and surrounding cells [40–42], it is conceivable that a Warburg-high subtype in the tumour centre could be to some part causally relevant for a lack of desmoplasia and/or intervening inflammatory infiltrate in SARIFA-positive cancers. Here, it has to be emphasised that this is speculative and that the exact underlying mechanism of SARIFA formation is currently unclear.

Moreover, emerging evidence indicates that cancer cells facilitate the dedifferentiation of adjacent adipocytes to form cancer-associated adipocytes (CAAs) [43], which, in turn, may provide metabolites to cancer cells to feed into the glycolytic pathway [35], highlighting the strong interrelationship between the Warburg-effect and alterations in lipid metabolism.

To further explore the relationship between SARIFA-status and Warburg-subtype, stratified survival analyses were performed. We only observed a significant association with survival for the Warburg-high subtype when SARIFA-status was unknown. These results suggest that SARIFA-status may be either a confounding factor or mediating factor explaining the association between Warburg-subtype and survival. As SARIFA-status and Warburgsubtype likely reflect different metabolic pathways (lipid metabolism and glycolysis), we hypothesized that a combination score of both metabolic pathways will have more prognostic power compared to the individual marker. However, our results indicate that the combined score did not improve patient prognostic stratification compared to using SARIFA-status alone. As Warburg-subtyping requires multiple immunohistochemical stainings, we believe that SARIFA alone is sufficient as H&E biomarker for prognosis estimation in the clinical routine. Previous studies suggested an association between SARIFA-positivity and other H&E biomarkers such as low proportionoftumor (i.e. high stromal content) [44] and non-mature desmoplastic reaction [45]. Combining these H&E-based biomarkers for survival analysis could be of interest for future studies. Whether such combination scores might enable a better prediction of response to certain therapies needs to be investigated in future studies.

Our findings could be of potential value for the development of novel drugs in CRC specifically targeting simultaneously both involved metabolic pathways. For example, Flaveny et al. [24] showed that the nuclear-receptor liver-X-receptor (LXR) agonist SR9243 inhibits both glycolysis and lipogenesis in cancer cells in vitro and in mice models of various cancer types including colon cancer.

Even though SARIFA-status and Warburg-subtype as metabolic biomarkers are measured at different locations within the tumour (SARIFA-status: invasion front; Warburg-subtyping: tissue microarray core, tumour centre), they show a clear association, suggestive of an overall altered, more aggressive tumour biology. Linking SARIFA-positivity (i.e. direct tumour-adipocyte interaction at the invasion front) to the Warburg-high subtype in the tumour centre indicates that SARIFA-positive CRCs are not only showing a different biological behaviour at the invasion front but also in the tumour centre, which is biologically interesting. This is in line with our findings that SARIFA-positive CRCs are characterised by a broad dysregulation of gene expression based on bulk RNAdata [46]. Based on our findings, further functional investigations (e.g. based on cell culture experiments) and/or spatially resolved molecular studies (e.g. single cell RNA profiling or spatial transcriptomics/proteomics assays) are necessary to better understand the role of Warburg-subtype in SARIFA-positive CRCs. Moreover, comparing IHC-based Warburg-subtypes between central tumour parts and the invasion front could be part of further studies.

### Strengths and limitations

Strengths of this study include the use of a large population-based series of incident CRC patients, the nearly complete follow-up, and the availability of tumour material for a large number of CRC patients. Despite this, the current study also has several limitations. First, Warburg-subtype and SARIFA-status were not determined on the same part of the tumour, as already described above. Warburg-subtype was determined on tissue microarray (TMA) cores that were taken from areas with the highest tumour density (i.e., centre of the tumour), whereas SARIFA-status was determined on whole tissue slides at the invasion front of the tumour. As a result, it is important to approach the results of this study with careful consideration. Second, we did not have access to a validation cohort to confirm the observed associations. Yet, our SARIFA-status findings are consistent with our initial discovery study, where an exploratory as well as a validation collective was analysed [9]. Third, as the frequency of SARIFA-positivity was associated with tumour location, more detailed information of tumour sidedness could be very interesting in this context. It is known that right sided colon cancer is more aggressive and biologically distinct [47], leading also to differences in treatment response between right and left sided colon cancer [48, 49]. Fourth, we did not adjust for multiple testing. Lastly, limitations with regard to Warburg-subtyping were described in detail previously [22].

## Conclusions

In this large prospective series of colorectal cancer (CRC) patients, we have shown that Stroma AReactive Invasion Front Areas (SARIFA)-status has prognostic value, independent of known prognostic factors such as pathological tumour-node-metastasis (pTNM) stage. Furthermore, our results indicate a potential relationship between SARIFA-status and Warburg-subtype. However, future large(r)-scale prospective studies are necessary to validate our results and further explore the relationship between Warburg-subtype and SARIFA-status as these could provide further insights how the Warburg-effect and lipid metabolism may interact with each other in cancer progression. Interfering with these metabolic alterations in CRC could potentially be a novel drug target. Our two biomarkers, SARIFA-status and Warburg-subtype, may be relevant in adequate patient selection, especially considering that they both rely on routine pathologic methods (H&E and IHC) and therefore would be relatively easy to implement in daily practice.

### Supplementary Information

Supplementary Material 1. 

## Data Availability

The datasets generated and/or analysed during the current study are not publicly available because the informed consent does not allow for that.

## Abbreviations

CRC: Colorectal cancer
CINSARC: Complexity INdex in SARComas
CI: Confidence interval
CMS: Consensus molecular subtypes
DL: Deep learning
PALGA: Dutch Pathology Registry
GLUT1: Glucose transporter 1
H&E: Haematoxylin & Eosin
HR: Hazard ratio
IHC: Immunohistochemistry
LDHA: Lactate dehydroganase A
METC: Medical Ethical Committee
MMR: Mismatch repair
dMMR: Deficient mismatch repair
pMMR: proficient mismatch repair
MCT4: Monocarboxylate transporter 4
MLH1: MutL Homolog 1
MSH2: MutS Homolog 2
NLCS: Netherlands Cohort Study
pTNM: Pathological tumour-node-metastasis
PTEN: Phosphatase and tensin homolog
PKM2: Pyruvate kinase M2
SARIFA: Stroma Areactive Invasion Front Areas
TNM: Tumour-node-metastasis
