# Supplementary material for: The relationship between Stroma AReactive Invasion Front Areas (SARIFA), Warburg-subtype and survival: results from a large prospective series of colorectal cancer patients
Source: Cancer Metab. 2024 Jul 11;12:21. doi: 10.1186/s40170-024-00349-z (PMC11241902; doi:10.1186/s40170-024-00349-z)
Supplement: Supplementary file 1 — Supplementary Material 1. [file 40170_2024_349_MOESM1_ESM.docx]

# SUPPLEMENTARY MATERIAL

**Supplementary Table S1 –** TNM classification of colorectal cancer, according to incidence year.

| Topography | 153.0-154.1 or C18-C20 | | |
| --- | --- | --- | --- |
| Histology | Epithelial cancers (M8010-8580) | | |
|  |  |  |  |
| **Incidence years** | **1988-2002** |  |  |
| **TNM versions** | **4.1-5** |  |  |
|  |  |  |  |
| Stage | T | N | M |
| I | 1-2 | 0/X | 0/X |
| II | 3-4 | 0/X | 0/X |
| III | Any T | 1-3 | 0/X |
| IV | Any T | Any N | 1 |
| X | X | 0/X | 0/X |
|  |  |  |  |
|  |  |  |  |
| **Incidence years** | **2003-2009** |  |  |
| **TNM versions** | **6** |  |  |
|  |  |  |  |
| Stage | T | N | M |
| I | 1-2 | 0/X | 0/X |
| IIA | 3 | 0/X | 0/X |
| IIB | 4 | 0/X | 0/X |
| III | X | 1 | 0/X |
| IIIA | 1-2 | 1 | 0/X |
| IIIB | 3-4 | 1 | 0/X |
| IIIC | Any T | 2 | 0/X |
| IV | Any T | Any N | 1 |
| X | X | 0/X | 0/X |

**Supplementary Table S2 -** Clinical and molecular characteristics of CRC patients with a known and unknown SARIFA-status.

|  | | **SARIFA-unknown (*n* = 524)** | **SARIFA-known (*n* = 1,727)** | ***p-*value^1^** |
| --- | --- | --- | --- | --- |
|  | |  |  |  |
|  | |  |  |  |
| **Age at diagnosis in years, median (range)** | | 74.0 (56.0-89.0) | 74.0 (55.0-89.0) | 0.981^2^ |
| **Sex, *n* (%)** | |  |  |  |
|  | Men | 284 (54.2) | 958 (55.5) | 0.608 |
|  | Women | 240 (45.8) | 769 (44.5) |  |
| **Tumor location, *n* (%)** | |  |  |  |
|  | Colon | 413 (78.8) | 1288 (74.6) | 0.142 |
|  | Rectosigmoid | 45 (8.6) | 177 (10.3) |  |
|  | Rectum | 66 (12.6) | 262 (15.2) |  |
| **pTNM stage, *n* (%)** | |  |  |  |
|  | I | 109 (20.8) | 315 (18.2) | 0.476 |
|  | II | 196 (37.4) | 672 (38.9) |  |
|  | III | 128 (24.4) | 454 (26.3) |  |
|  | IV | 80 (15.3) | 245 (14.2) |  |
|  | Unknown | 11 (2.1) | 41 (2.4) |  |
| **Tumor extension (pT), *n* (%)** | |  |  |  |
|  | T1 | 30 (5.7) | 65 (4.8) | 0.103 |
|  | T2 | 100 (19.1) | 306 (17.7) |  |
|  | T3 | 319 (60.9) | 1138 (65.9) |  |
|  | T4 | 59 (11.3) | 174 (10.1) |  |
|  | Unknown | 16 (3.1) | 44 (2.6) |  |
| **Lymph node involvement (pN), *n* (%)** | |  |  |  |
|  | N0 | 274 (52.3) | 900 (52.1) | 0.903 |
|  | N+ | 189 (36.1) | 629 (36.4) |  |
|  | Unknown | 61 (11.6) | 198 (11.5) |  |
| **Differentiation grade, *n* (%)** | |  |  |  |
|  | Well | 49 (9.4) | 152 (8.8) | 0.686 |
|  | Moderate | 340 (64.9) | 1138 (65.9) |  |
|  | Poor/undifferentiated | 85 (16.2) | 312 (18.1) |  |
|  | Unknown | 50 (9.5) | 125 (7.2) |  |
| **Adjuvant therapy, *n* (%)** | |  |  |  |
|  | No | 448 (85.5) | 1426 (82.6) | 0.203 |
|  | Yes | 74 (14.1) | 282 (16.3) |  |
|  | Unknown | 2 (0.4) | 19 (1.1) |  |
| **MMR status, *n* (%)** | |  |  |  |
|  | Proficient | 438 (83.6) | 1537 (89.0) | 0.329 |
|  | Deficient | 63 (12.0) | 190 (11.0) |  |
|  | Unknown | 23 (4.4) | - |  |
| **Warburg-subtypes** | |  |  |  |
|  | Warburg-low | 159 (30.3) | 493 (28.6) | 0.683 |
|  | Warburg-moderate | 180 (34.4) | 622 (36.0) |  |
|  | Warburg-high | 185 (35.3) | 612 (35.4) |  |
| ^1^*P*-value for the Chi-square test, unless otherwise specified. Presented *p*-values exclude the ‘unknown’ category for pTNM stage, pT, pN, differentiation grade, adjuvant therapy, and MMR status.  ^2^*P*-value for the Kruskal-Wallis test. | | | | |

**Supplementary Table S3** – Univariable and multivariable-adjusted hazard ratios (HRs) and 95% confidence intervals (CIs) for associations between SARIFA status and CRC-specific and overall survival of colorectal cancer patients (*n* = 2,251) within the Netherlands Cohort Study (NLCS, 1986-2006), according to tumor location (colon, rectosigmoid, rectum).

|  | | **N** |  | **CRC-specific survival** | | |  | **Overall survival** | | |
| --- | --- | --- | --- | --- | --- | --- | --- | --- | --- | --- |
|  |  |  |  | **CRC deaths (%)** | **HR (95% CI)** | |  | **Deaths (%)** | **HR (95% CI)** | |
|  | |  |  |  | **Univariable** | **Multivariable-adjusted** |  |  | **Univariable** | **Multivariable-adjusted** |
| **Colorectal** | |  |  |  |  |  |  |  |  |  |
|  | SARIFA-negative | 1231 |  | 406 (33.0) | 1.00 (ref) | 1.00 (ref) |  | 726 (59.0) | 1.00 (ref) | 1.00 (ref) |
|  | SARIFA-positive | 496 |  | 317 (63.9) | 2.75 (2.37-3.19) | 1.67 (1.43-1.95) |  | 402 (81.0) | 2.09 (1.85-2.37) | 1.49 (1.30-1.69) |
|  | SARIFA-unknown | 524 |  | 210 (40.1) | 1.25 (1.15-1.60) | 1.22 (1.03-1.45) |  | 335 (63.9) | 1.22 (1.07-1.39) | 1.15 (1.01-1.32) |
| **Colon** | |  |  |  |  |  |  |  |  |  |
|  | SARIFA-negative | 863 |  | 284 (32.9) | 1.00 (ref) | 1.00 (ref) |  | 514 (59.6) | 1.00 (ref) | 1.00 (ref) |
|  | SARIFA-positive | 425 |  | 273 (64.2) | 2.73 (2.31-3.23) | 1.75 (1.47-2.08) |  | 342 (80.5) | 2.03 (1.77-2.33) | 1.54 (1.33-1.79) |
|  | SARIFA-unknown | 413 |  | 157 (38.0) | 1.27 (1.05-1.55) | 1.13 (0.93-1.38) |  | 264 (63.9) | 1.20 (1.03-1.39) | 1.11 (0.96-1.30) |
| **Rectosigmoid** | |  |  |  |  |  |  |  |  |  |
|  | SARIFA-negative | 142 |  | 37 (26.1) | 1.00 (ref) | 1.00 (ref) |  | 86 (60.6) | 1.00 (ref) | 1.00 (ref) |
|  | SARIFA-positive | 35 |  | 21 (60.0) | 3.59 (2.09-6.16) | 2.31 (1.27-4.20) |  | 31 (88.6) | 2.49 (1.64-3.77) | 1.61 (1.01-2.55) |
|  | SARIFA-unknown | 45 |  | 24 (53.3) | 2.46 (1.47-4.11) | 3.58 (2.01-6.36) |  | 30 (66.7) | 1.33 (0.88-2.02) | 1.85 (1.19-2.90) |
| **Rectum** | |  |  |  |  |  |  |  |  |  |
|  | SARIFA-negative | 226 |  | 85 (37.6) | 1.00 (ref) | 1.00 (ref) |  | 126 (55.8) | 1.00 (ref) | 1.00 (ref) |
|  | SARIFA-positive | 36 |  | 23 (63.9) | 2.28 (1.44-3.62) | 1.09 (0.66-1.79) |  | 29 (80.6) | 2.01 (1.34-3.01) | 1.06 (0.68-1.63) |
|  | SARIFA-unknown | 66 |  | 29 (43.9) | 1.29 (0.84-1.96) | 1.20 (0.78-1.84) |  | 41 (62.1) | 1.24 (0.87-1.76) | 1.17 (0.81-1.69) |
| CRC, colorectal cancer; HR, hazard ratio; CI, confidence interval.  ^1^Multivariable-adjusted model included SARIFA status (positive, negative, unknown), age at diagnosis (years), sex (men, women),pTNM stage (I, II, III, IV, unknown), differentiation grade (well, moderate, poor/undifferentiated, unknown), adjuvant therapy (no, yes, unknown), and MMR deficiency (no, yes, unknown). | | | | | | | | | | |

**Supplementary Table S4** - Univariable and multivariable-adjusted hazard ratios (HRs) and 95% confidence intervals (CIs) for associations between SARIFA status and CRC-specific and overall survival of colorectal cancer patients (*n* = 2,251) within the Netherlands Cohort Study (NLCS, 1986-2006), according to pTNM stage (I, II, III, IV).

|  | | **N** |  | **CRC-specific survival** | | |  | **Overall survival** | | |
| --- | --- | --- | --- | --- | --- | --- | --- | --- | --- | --- |
|  |  |  |  | **CRC deaths (%)** | **HR (95% CI)** | |  | **Deaths (%)** | **HR (95% CI)** | |
|  | |  |  |  | **Univariable** | **Multivariable-adjusted** |  |  | **Univariable** | **Multivariable-adjusted** |
| **Colorectal** | |  |  |  |  |  |  |  |  |  |
|  | SARIFA-negative | 1231 |  | 406 (33.0) | 1.00 (ref) | 1.00 (ref) |  | 726 (59.0) | 1.00 (ref) | 1.00 (ref) |
|  | SARIFA-positive | 496 |  | 317 (63.9) | 2.75 (2.37-3.19) | 1.67 (1.43-1.95) |  | 402 (81.0) | 2.09 (1.85-2.37) | 1.49 (1.30-1.69) |
|  | SARIFA-unknown | 524 |  | 210 (40.1) | 1.25 (1.15-1.60) | 1.22 (1.03-1.45) |  | 335 (63.9) | 1.22 (1.07-1.39) | 1.15 (1.01-1.32) |
| **pTNM stage I** | |  |  |  |  |  |  |  |  |  |
|  | SARIFA-negative | 309 |  | 46 (14.9) | 1.00 (ref) | 1.00 (ref) |  | 145 (46.9) | 1.00 (ref) | 1.00 (ref) |
|  | SARIFA-positive | 6 |  | 3 (50.0) | 5.81 (1.90-18.72) | 8.87 (2.41-32.71) |  | 4 (66.7) | 2.87 (1.06-7.75) | 4.40 (1.51-12.77) |
|  | SARIFA-unknown | 109 |  | 18 (16.5) | 1.11 (0.64-1.92) | 1.18 (0.68-2.05) |  | 45 (41.3) | 0.88 (0.63-1.23) | 0.88 (0.62-1.24) |
| **pTNM stage II** | |  |  |  |  |  |  |  |  |  |
|  | SARIFA-negative | 514 |  | 119 (23.2) | 1.00 (ref) | 1.00 (ref) |  | 270 (52.5) | 1.00 (ref) | 1.00 (ref) |
|  | SARIFA-positive | 158 |  | 63 (39.9) | 1.89 (1.39-2.57) | 1.84 (1.34-2.51) |  | 102 (64.6) | 1.40 (1.11-1.76) | 1.45 (1.14-1.82) |
|  | SARIFA-unknown | 196 |  | 55 (28.1) | 1.30 (0.95-1.79) | 1.19 (0.85-1.66) |  | 113 (57.7) | 1.19 (0.95-1.48) | 1.18 (0.94-1.48) |
| **pTNM stage III** | |  |  |  |  |  |  |  |  |  |
|  | SARIFA-negative | 277 |  | 136 (49.1) | 1.00 (ref) | 1.00 (ref) |  | 192 (69.3) | 1.00 (ref) | 1.00 (ref) |
|  | SARIFA-positive | 177 |  | 107 (60.5) | 1.55 (1.20-1.99) | 1.56 (1.20-2.02) |  | 142 (80.2) | 1.48 (1.19-1.84) | 1.48 (1.18-1.85) |
|  | SARIFA-unknown | 128 |  | 63 (49.2) | 1.07 (0.80-1.45) | 1.18 (0.87-1.61) |  | 91 (71.1) | 1.10 (0.86-1.41) | 1.21 (0.93-1.57) |
| **pTNM stage IV** | |  |  |  |  |  |  |  |  |  |
|  | SARIFA-negative | 103 |  | 95 (92.2) | 1.00 (ref) | 1.00 (ref) |  | 103 (100.0) | 1.00 (ref) | 1.00 (ref) |
|  | SARIFA-positive | 142 |  | 131 (92.3) | 1.56 (1.19-2.04) | 1.65 (1.25-2.18) |  | 141 (99.3) | 1.55 (1.20-2.01) | 1.67 (1.28-2.18) |
|  | SARIFA-unknown | 80 |  | 70 (87.5) | 1.22 (0.90-1.67) | 1.40 (1.02-1.92) |  | 79 (98.8) | 1.26 (0.94-1.69) | 1.43 (1.06-1.93) |
| CRC, colorectal cancer; HR, hazard ratio; CI, confidence interval.  ^1^Multivariable-adjusted model included SARIFA status (positive, negative, unknown), age at diagnosis (years), sex (men, women), tumor location (colon, rectosigmoid, rectum), differentiation grade (well, moderate, poor/undifferentiated, unknown), adjuvant therapy (no, yes, unknown), and MMR deficiency (no, yes, unknown). | | | | | | | | | | |

**Supplementary Table S5 -** Univariable and multivariable-adjusted hazard ratios (HRs) and 95% confidence intervals (CIs) for associations between SARIFA status and CRC-specific and overall survival of colorectal cancer patients (*n* = 2,251) within the Netherlands Cohort Study (NLCS, 1986-2006), according to Warburg-subtype (Warburg-low, Warburg-moderate, Warburg-high).

|  | | **N** |  | **CRC-specific survival** | | |  | **Overall survival** | | |
| --- | --- | --- | --- | --- | --- | --- | --- | --- | --- | --- |
|  |  |  |  | **CRC deaths (%)** | **HR (95% CI)** | |  | **Deaths (%)** | **HR (95% CI)** | |
|  | |  |  |  | **Univariable** | **Multivariable-adjusted** |  |  | **Univariable** | **Multivariable-adjusted** |
| **Colorectal** | |  |  |  |  |  |  |  |  |  |
|  | SARIFA-negative | 1231 |  | 406 (33.0) | 1.00 (ref) | 1.00 (ref) |  | 726 (59.0) | 1.00 (ref) | 1.00 (ref) |
|  | SARIFA-positive | 496 |  | 317 (63.9) | 2.75 (2.37-3.19) | 1.67 (1.43-1.95) |  | 402 (81.0) | 2.09 (1.85-2.37) | 1.49 (1.30-1.69) |
|  | SARIFA-unknown | 524 |  | 210 (40.1) | 1.25 (1.15-1.60) | 1.22 (1.03-1.45) |  | 335 (63.9) | 1.22 (1.07-1.39) | 1.15 (1.01-1.32) |
| **Warburg-low** | |  |  |  |  |  |  |  |  |  |
|  | SARIFA-negative | 380 |  | 121 (31.8) | 1.00 (ref) | 1.00 (ref) |  | 218 (57.4) | 1.00 (ref) | 1.00 (ref) |
|  | SARIFA-positive | 113 |  | 70 (61.9) | 2.59 (1.93-3.49) | 1.76 (1.28-2.41) |  | 88 (77.9) | 1.94 (1.51-2.49) | 1.58 (1.21-2.06) |
|  | SARIFA-unknown | 159 |  | 54 (34.0) | 1.15 (0.83-1.58) | 1.02 (0.73-1.43) |  | 96 (60.4) | 1.14 (0.89-1.45) | 1.04 (0.81-1.34) |
| **Warburg-moderate** | |  |  |  |  |  |  |  |  |  |
|  | SARIFA-negative | 458 |  | 156 (34.1) | 1.00 (ref) | 1.00 (ref) |  | 269 (58.7) | 1.00 (ref) | 1.00 (ref) |
|  | SARIFA-positive | 164 |  | 114 (69.5) | 3.19 (2.50-4.08) | 1.88 (1.45-2.44) |  | 136 (82.9) | 2.40 (1.94-2.95) | 1.58 (1.26-1.97) |
|  | SARIFA-unknown | 180 |  | 69 (38.3) | 1.22 (0.92-1.61) | 1.12 (0.84-1.49) |  | 109 (60.6) | 1.12 (0.89-1.40) | 1.04 (0.83-1.31) |
| **Warburg-high** | |  |  |  |  |  |  |  |  |  |
|  | SARIFA-negative | 393 |  | 129 (32.8) | 1.00 (ref) | 1.00 (ref) |  | 239 (60.8) | 1.00 (ref) | 1.00 (ref) |
|  | SARIFA-positive | 219 |  | 133 (60.7) | 2.52 (1.97-3.21) | 1.38 (1.06-1.79) |  | 178 (81.3) | 1.93 (1.58-2.34) | 1.30 (1.06-1.60) |
|  | SARIFA-unknown | 185 |  | 87 (47.0) | 1.70 (1.29-2.23) | 1.44 (1.08-1.91) |  | 130 (70.3) | 1.40 (1.13-1.73) | 1.32 (1.06-1.65) |
| CRC, colorectal cancer; HR, hazard ratio; CI, confidence interval.  ^1^Multivariable-adjusted model included SARIFA status (positive, negative, unknown), age at diagnosis (years), sex (men, women), tumor location (colon, rectosigmoid, rectum), pTNM stage (I, II, III, IV, unknown), differentiation grade (well, moderate, poor/undifferentiated, unknown), adjuvant therapy (no, yes, unknown), and MMR deficiency (no, yes, unknown). | | | | | | | | | | |

**Supplementary Table S6** - Univariable and multivariable-adjusted hazard ratios (HRs) and 95% confidence intervals (CIs) for associations between Warburg-subtypes and CRC-specific and overall survival of colorectal cancer patients (*n* = 2,251) within the Netherlands Cohort Study (NLCS, 1986-2006), according to SARIFA-status (SARIFA-negative, SARIFA-positive, SARIFA-unknown).

|  | | **N** |  | **CRC-specific survival** | | |  | **Overall survival** | | |
| --- | --- | --- | --- | --- | --- | --- | --- | --- | --- | --- |
|  |  |  |  | **CRC deaths (%)** | **HR (95% CI)** | |  | **Deaths (%)** | **HR (95% CI)** | |
|  | |  |  |  | **Univariable** | **Multivariable-adjusted** |  |  | **Univariable** | **Multivariable-adjusted** |
| **Colorectal** | |  |  |  |  |  |  |  |  |  |
|  | Warburg-low | 652 |  | 245 (37.6) | 1.00 (ref) | 1.00 (ref) |  | 402 (61.7) | 1.00 (ref) | 1.00 (ref) |
|  | Warburg-moderate | 802 |  | 339 (42.2) | 1.16 (0.98-1.36) | 1.04 (0.88-1.22) |  | 514 (64.1) | 1.07 (0.94-1.22) | 1.01 (0.89-1.15) |
|  | Warburg-high | 797 |  | 349 (43.8) | 1.27 (1.08-1.50) | 1.17 (0.99-1.38) |  | 547 (68.6) | 1.24 (1.09-1.41) | 1.18 (1.03-1.34) |
| **SARIFA-negative** | |  |  |  |  |  |  |  |  |  |
|  | Warburg-low | 380 |  | 121 (31.8) | 1.00 (ref) | 1.00 (ref) |  | 218 (57.4) | 1.00 (ref) | 1.00 (ref) |
|  | Warburg-moderate | 458 |  | 156 (34.1) | 1.07 (0.84-1.35) | 0.98 (0.77-1.24) |  | 269 (58.7) | 1.02 (0.85-1.22) | 0.99 (0.83-1.19) |
|  | Warburg-high | 393 |  | 129 (32.8) | 1.07 (0.83-1.37) | 1.10 (0.86-1.42) |  | 239 (60.8) | 1.11 (0.92-1.33) | 1.12 (0.93-1.35) |
| **SARIFA-positive** | |  |  |  |  |  |  |  |  |  |
|  | Warburg-low | 113 |  | 70 (61.9) | 1.00 (ref) | 1.00 (ref) |  | 88 (77.9) | 1.00 (ref) | 1.00 (ref) |
|  | Warburg-moderate | 164 |  | 114 (69.5) | 1.31 (0.98-1.77) | 1.03 (0.76-1.40) |  | 136 (82.9) | 1.26 (0.97-1.65) | 1.02 (0.78-1.34) |
|  | Warburg-high | 219 |  | 133 (60.7) | 1.05 (0.79-1.41) | 0.91 (0.68-1.23) |  | 178 (81.3) | 1.12 (0.87-1.45) | 0.99 (0.77-1.29) |
| **SARIFA-unknown** | |  |  |  |  |  |  |  |  |  |
|  | Warburg-low | 159 |  | 54 (34.0) | 1.00 (ref) | 1.00 (ref) |  | 96 (60.4) | 1.00 (ref) | 1.00 (ref) |
|  | Warburg-moderate | 180 |  | 69 (38.3) | 1.13 (0.79-1.61) | 1.11 (0.77-1.61) |  | 109 (60.6) | 1.00 (0.76-1.32) | 1.01 (0.77-1.34) |
|  | Warburg-high | 185 |  | 87 (47.0) | 1.57 (1.12-2.21) | 1.60 (1.12-2.27) |  | 130 (70.3) | 1.35 (1.04-1.76) | 1.41 (1.07-1.85) |
| CRC, colorectal cancer; HR, hazard ratio; CI, confidence interval. | | | | | | | | | | |
